# SFXN3 is a Prognostic Marker and Promotes the Growth of Acute Myeloid Leukemia

**DOI:** 10.1007/s12013-024-01326-5

**Published:** 2024-06-14

**Authors:** Fengbo Jin, Limei He, Jing Wang, Yu Zhang, Mingzhen Yang

**Affiliations:** 1 Department of Hematology, the First Affiliated Hospital of Anhui Medical University, Hefei, 230000 China; 2 Anhui Public Health Clinical Center, Hefei, China

**Keywords:** Acute myeloid leukemia, SFXN3, NFKB1, CCND1

## Abstract

Acute myeloid leukemia (AML) is a heterogeneous disease with rapid progression and frequent mutations. Sideroflexin3 (SFXN3) has been shown to be involved in various neurodegenerative diseases. However, the role of SFXN3 in AML remains unclear. The level and prognostic value of SFXN3 were assessed in pan-cancer, especially AML, based on the data obtained from the TCGA database. The effect and mechanism of SFXN3 in AML were measured by fluorescence-activated cell sorting (FACS), qRT-PCR, western blotting in vitro and in vivo. The correlation between SFXN3 and the infiltration of immune cells in AML was assessed via cibersort and ssGSEA analyses. SFXN3 is expressed at higher levels in AML, and high SFXN3 level is associated with decreased overall survival rate (OSR) in AML. Next, knockdown of SFXN3 results in enhanced cell apoptosis and dropped cell proliferation. Then, knockdown of SFXN3 caused a reduction in the expression of CyclinD1 (CCND1) and nuclear factor of kappa light polypeptide gene enhancer in B-cells 1 (NFKB1). Finally, SFXN3 may related to the immunosuppressive state of AML. Increased SFXN3 expression is detected in AML, which indicates a poor prognosis and may link to immunosuppressive state of AML. In addition, SFXN3 can inhibit AML cells apoptosis and promote cell proliferation via enhancing CCND1 and NFKB1 levels.

## Introduction

Acute myeloid leukemia (AML), one of the most common malignancy of the blood system in adults, is a hematopoitic stem/progenitor cell malignant clonal disease [1, 2]. AML is a heterogeneous disease with rapid progression and frequent mutation [3]. In the United States, the five year overall survival rate (OSR) is only about 24%. Meanwhile, the AML patients died accounted for 62% among the leukemia related deaths [4]. Despite advances in anti-leukemia therapy, OSR has not been significantly improved due to the complex tumor micro-environment and stubborn mutation mechanisms [5]. Therefore, it is necessary to further explore the pathogenesis of AML and search for new biomarkers, so as to provide a hopeful perspective for the treatment of AML.

Sideroflexins (SLC56 family), mainly in eukaryotes, are highly conserved multi-transmembrane proteins inserted in the inner mitochondrial membrane and are considered to be metabolite transporters that may require the use of iron in mitochondria [6]. The Sideroflexins family has five homologs in humans (SFXN1-5), which are expressed differently in different tissues [7–9]. SFXN3 is a mitochondrial protein enriched in mature neurons and expressed developmentally [10]. Currently, SFXN3 has been shown to be involved in various neurodegenerative diseases, such as Parkinson’s disease and Alzheimer’s disease, through synaptic structures [11, 12]. SFXN3 Gene, homologous to SFXN1 Gene, a mitochondrial serine transporter, mediates serine entry into mitochondria and is an important step in the single carbon metabolic pathway [6]. Serine in mitochondria is converted to glycine and formate [8], and the growth and proliferation of many cancer cells is highly dependent on serine metabolism [13, 14]. Serum anti-SFXN3 levels in oral squamous cell carcinoma were slightly correlated with primary tumor size, and changes in serum anti-SFXN3 levels after treatment were correlated with clinical tumor load. As a potential tumor marker [15]. However, the role of SFXN3 in AML remains unclear. Therefore, it is very important to explore the biomarker value and prognostic value of SFXN3 in AML patients.

In this study, we firstly explored the expression and prognostic value of SFXN3 in AML. Then, we analyzed the function and mechanism of SFXN3 in AML. Finally, we examine the correlation between SFXN3 and the infiltration and activation of immune cells in AML.

## Results

### The Expression and Prognostic Value of SFXN3 in Pan-cancer, Especially in AML

**Fig. 1:**
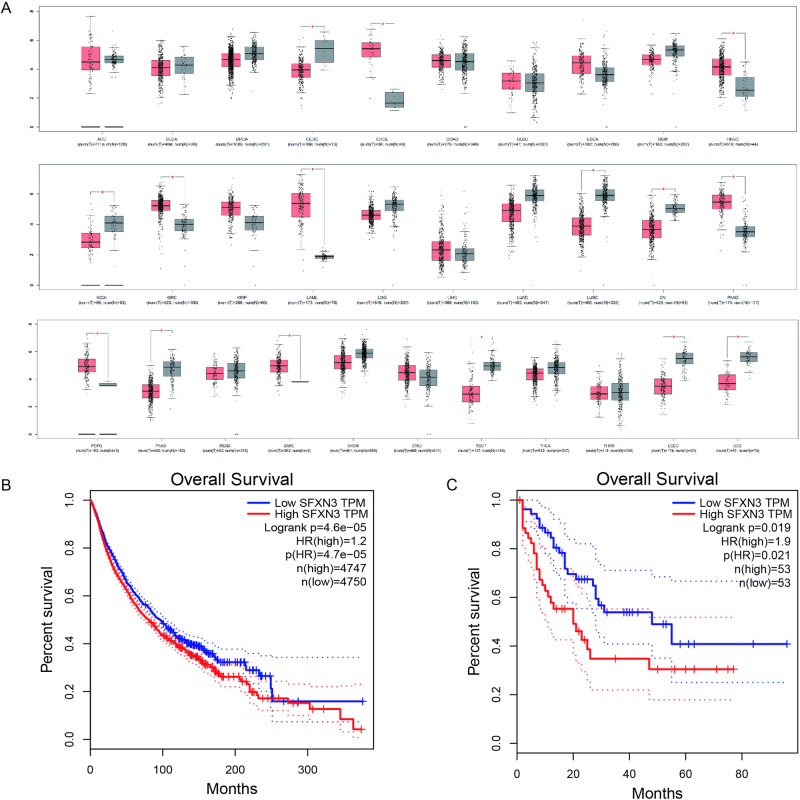
The expression and prognostic value of SFXN3 in Pan-cancer, Especially in AML. **A** The expression blots for SFXN3 from the GEPIA database in pancarcinoma. The red node represents tumor samples, and gray node represented normal samples. **B** OS for SFXN3 from the GEPIA database in pancarcinoma patients by median cut-offs. **C** OS for SFXN3 from the GEPIA database in AML patients by median cut-offs. OS overall survival

The level of SFXN3 in 31 types of pan-cancer was identified by GEPIA (Fig. 1A). Abnormal SFNX3 level was found in 15 tumors. Among which, increased SFXN3 levels were detected in 7 types of cancers, including AML (also named LAML in TCGA database), cholangiocarcinoma (CHOL), head and neck squamous cell carcinoma (HNSC), etc., while decreased SFXN3 levels were found in 8 types of cancers, including cervical and endocervical cancer (CESC), lung squamous cell carcinoma (LUSC), prostate adenocarcinoma (PRAD), etc. Interestingly, among these cancers with over-expressed SFXN3, AML exhibited the most dramatic up-regulation of SFXN3 expression compared with normal tissues. Further, survival curves were plotted using GEPIA to investigate the prognostic value of SFXN3 in pan-cancer (9497 samples) (Fig. 1B). Patients exhibiting high SFXN3 expression (4747 samples) are more likely to possess a higher overall survival rate (OSR) compared with those with low SFXN3 level (4750 samples). It is worth noting that high SFXN3 expression levels were also associated with decreased OSR in AML (Fig. 1C). Taken together, SFXN3 expression enhanced in AML and increased SFXN3 may indicated a poor prognosis.

### Silencing SFXN3 Promotes AML Apoptosis and Inhibits AML Proliferation In Vitro

**Fig. 2:**
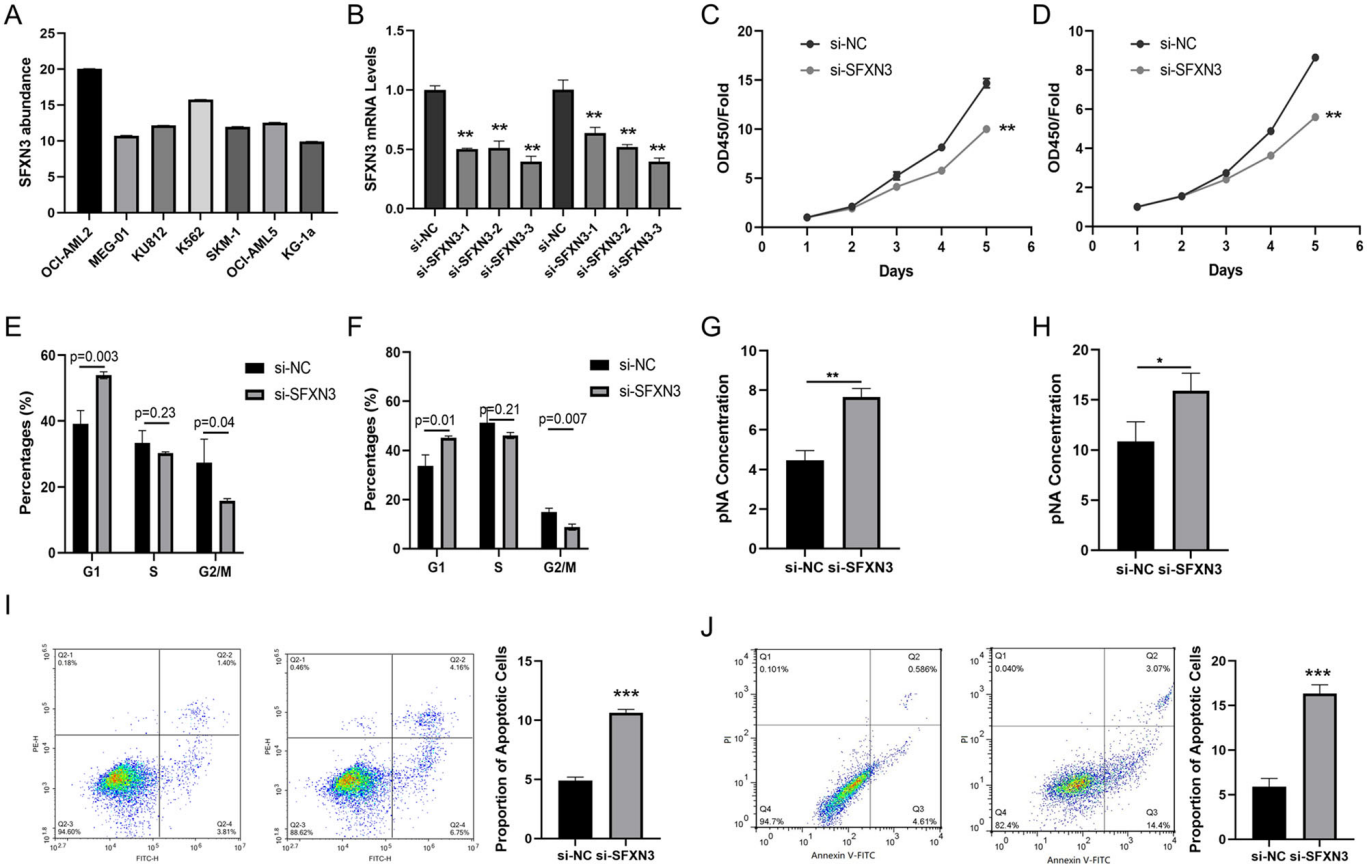
Silencing SFXN3 promotes AML apoptosis and inhibits AML proliferation in vitro. **A** The abundance of SFXN3 in AML cells measured by qRT-PCR. **B** The efficiency of siRNA targeting SFXN3 in AML cells measured by qRT-PCR. The proliferation ability of SKM-1 (**C**) and MEG-01 (**D**) cells measured by CCK-8 assay. The cell cycle of SKM-1 (**E**) and MEG-01 (**F**) cells measured by FACS. The activity of caspase-3 in SKM-1 (**G**) and MEG-01 (**H**) cells. The cell apoptosis of SKM-1 (**I**) and MEG-01 (**J**) cells measured by FACS. vs. si-NC group, *P < 0.05, **P < 0.01, ***P < 0.001

The enhanced SFXN3 in AML suggest that it may be an oncogene. To verify this, we firstly measured its levels in 7 AML cells in order to screen out tool cells for study. Through qRT-PCR, we found that the abundance of SFXN3 in all these cells were rich, especially in SKM-1 and MEG-01 cells (Fig. 2A). Then, we constructed siRNA targeting SFXN3 to knockdown its expression in SKM-1, KG-1a and MEG-01 cells. The results showed a significant reduction in SFXN3 expression in both cells treated with si-SFXN3, especially si-SFXN3-3 (Fig. 2B). As a result, we applied SKM-1 and MEG-01 cells as tool cells and si-SFXN3-3 as a tool to silence SFXN3. First, we explored the effect of SFXN3 on AML cell proliferation. The results of CCK-8 assay indicated that decreased SFXN3 results in reduced cell proliferation ability in both SKM-1 and MEG-01 cells (Fig. 2C, D). Similarly, the results of flow cell cycle experiments indicated that knockdown SFXN3 induce an arrest in G1/S stage (Fig. 2E, F). Then, we analyzed the effect of SFXN3 on caspase3 in AML cells. The results of caspase3 confirmed that decreased SFXN3 levels trigger the cleavage of caspase3 (Fig. 2G, H). Finally, the results of flow cell apoptosis assay demonstrated that silencing SFXN3 in AML cells lead to enhanced cell apoptosis in both SKM-1 and MEG-01 cells (Fig. 2I–J). Therefore, silencing SFXN3 in AML cells can cause decreased cell proliferation and increased cell apoptosis.

### Silencing SFXN3 Decreased the Growth of AML In Vivo

**Fig. 3:**
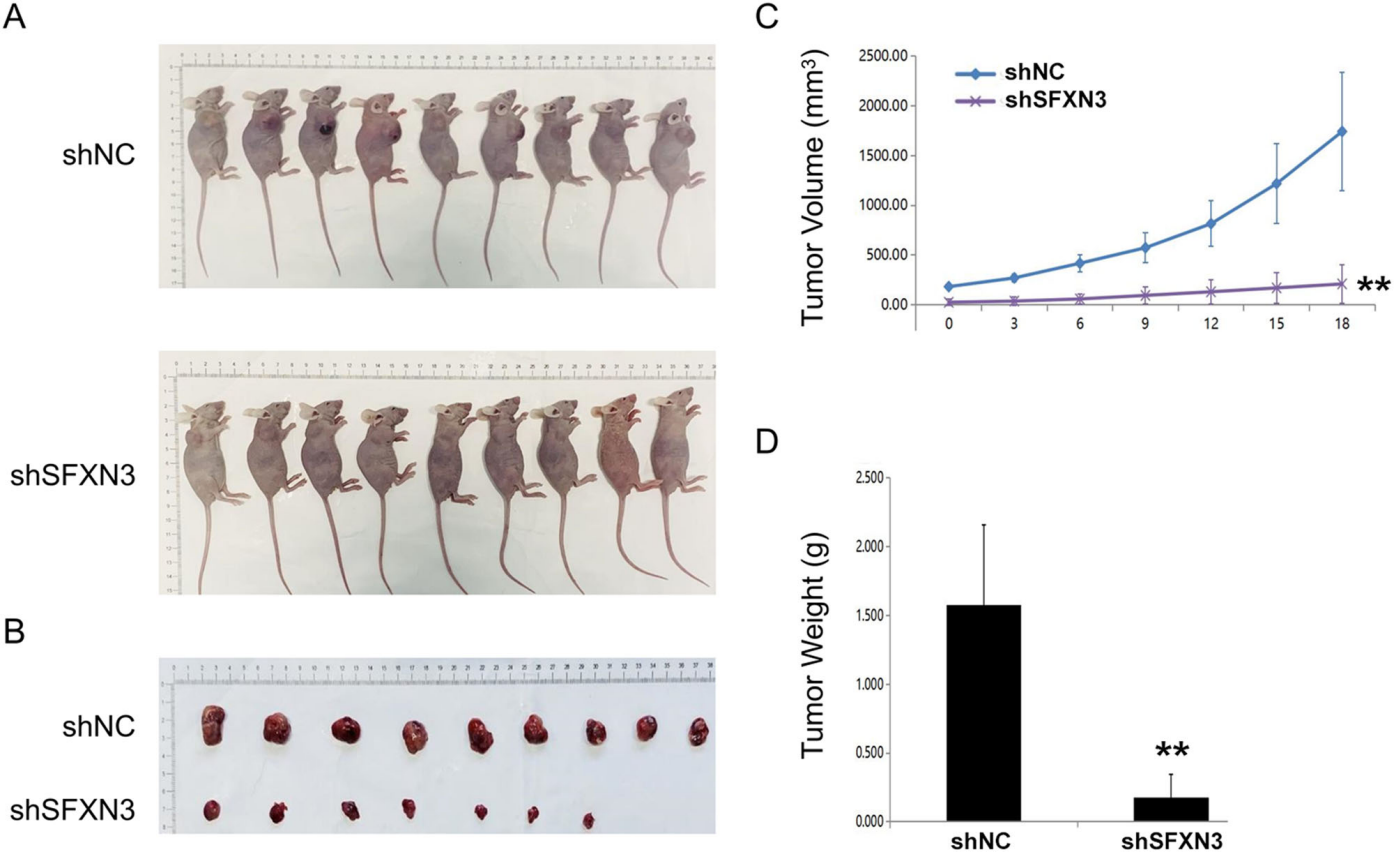
Silencing SFXN3 decreased the growth of AML in vivo. **A** The general view of the mice bearing AML. **B** The general view of the tumors. **C** The volume of the tumors. **D** The weight of the tumors. vs. shNC group, **P < 0.01

Above in vitro assays concluded the function of SFXN3 in AML proliferation and apoptosis. Hence, we further validated this in vivo. First, the graphs of the tumors exhibited that the tumors in the NC group are generally larger than those in the SFXN3 silencing group (Fig. 3A, B). Also, through analyzing the volume of the tumors measured every three days, we found that the tumor volume increase much quicker in the NC group (Fig. 3C). Finally, the tumors were weighted, and we found that the tumor weight in the experimental group is lighter than that in the NC group (Fig. 3D). Taken together, silencing SFXN3 cause an inhibition of tumor growth.

### The Mechanism of SFXN3 in Regulating AML Growth

**Fig. 4:**
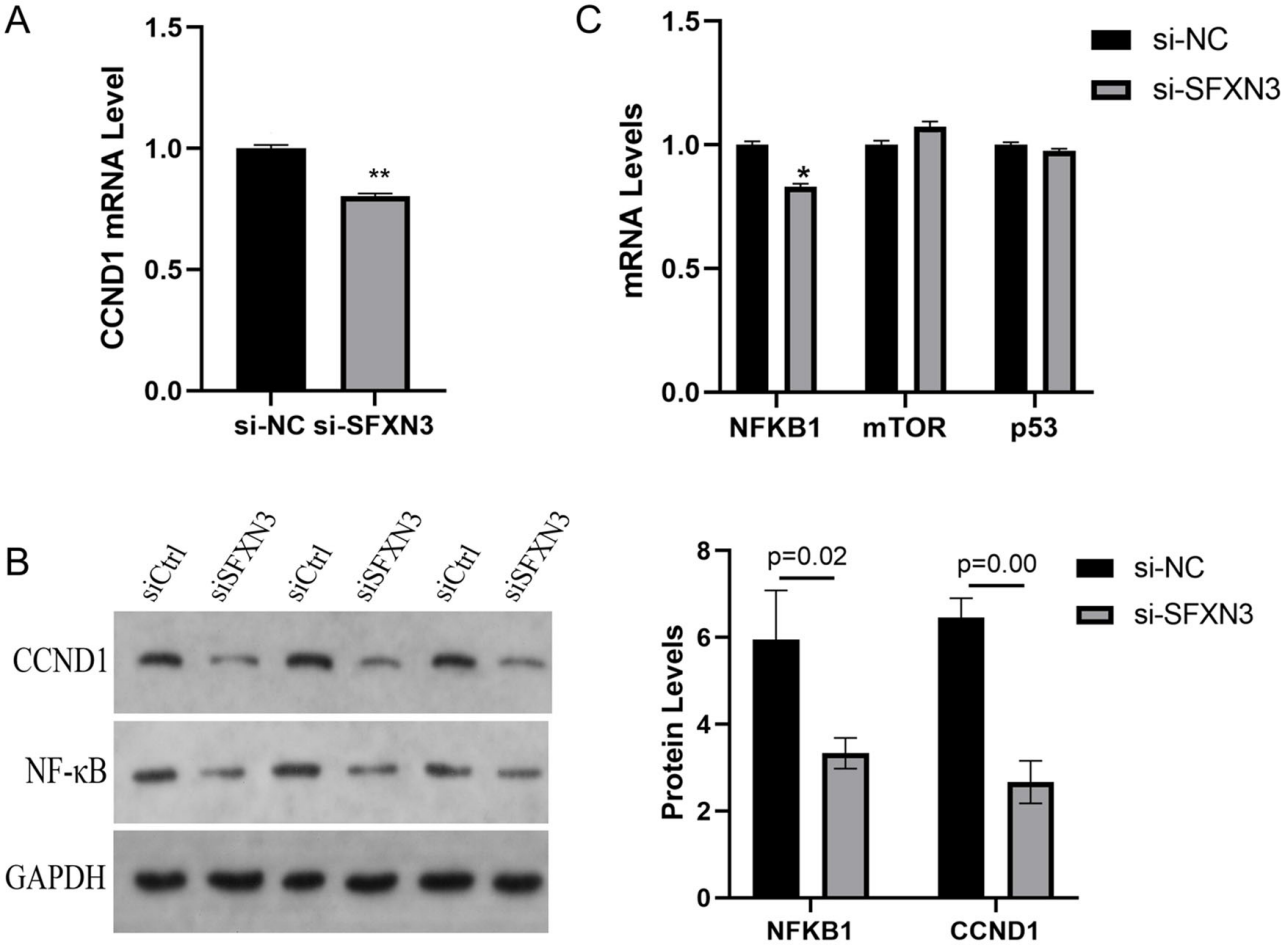
The mechanism of SFXN3 in regulating AML growth. **A** The level of CCND1 in MEG-01 cells measured by qRT-PCR. **B** The level of CCND1 in MEG-01 cells measured by western blotting. **C** The level of NFKB1, p53, mTOR in MEG-01 cells measured by qRT-PCR. D: The level of NFKB1 in MEG-01 cells measured by western blotting. vs. si-NC group, *P < 0.05, ** P < 0.01

As described above, SFXN3 induce G1/S arrest of AML cells, therefore we hypothesized that SFXN3 may regulates the expression of CCND1. To validate our suggestion, we measured the level of CCND1 in MEG-01 cells with or without SFXN3 silencing. The results of qRT-PCR demonstrated that the levels of CCND1 dropped in shSFXN3 MEG-01 cells (Fig. 4A). Further western blotting revealed that the level of CCND1 protein reduced after knocking down SFXN3 (Fig. 4B). Previous studies reported that CCND1 was regulated by NF-κB1, p53, mTOR and etc. Thus, we selected some of these markers for further detection. The results of qRT-PCR demonstrated that only the level of NF-κB1 decrease in shSFXN3 AML cells (Fig. 4C), which has been validated by further western blotting (Fig. 4B). Therefore, SFXN3 may promote the proliferation of AML cells via NFKB1 activated CCND1-CDK4 signaling.

### SFXN3 is Linked to Immune Cell Infiltration in AML

**Fig. 5:**
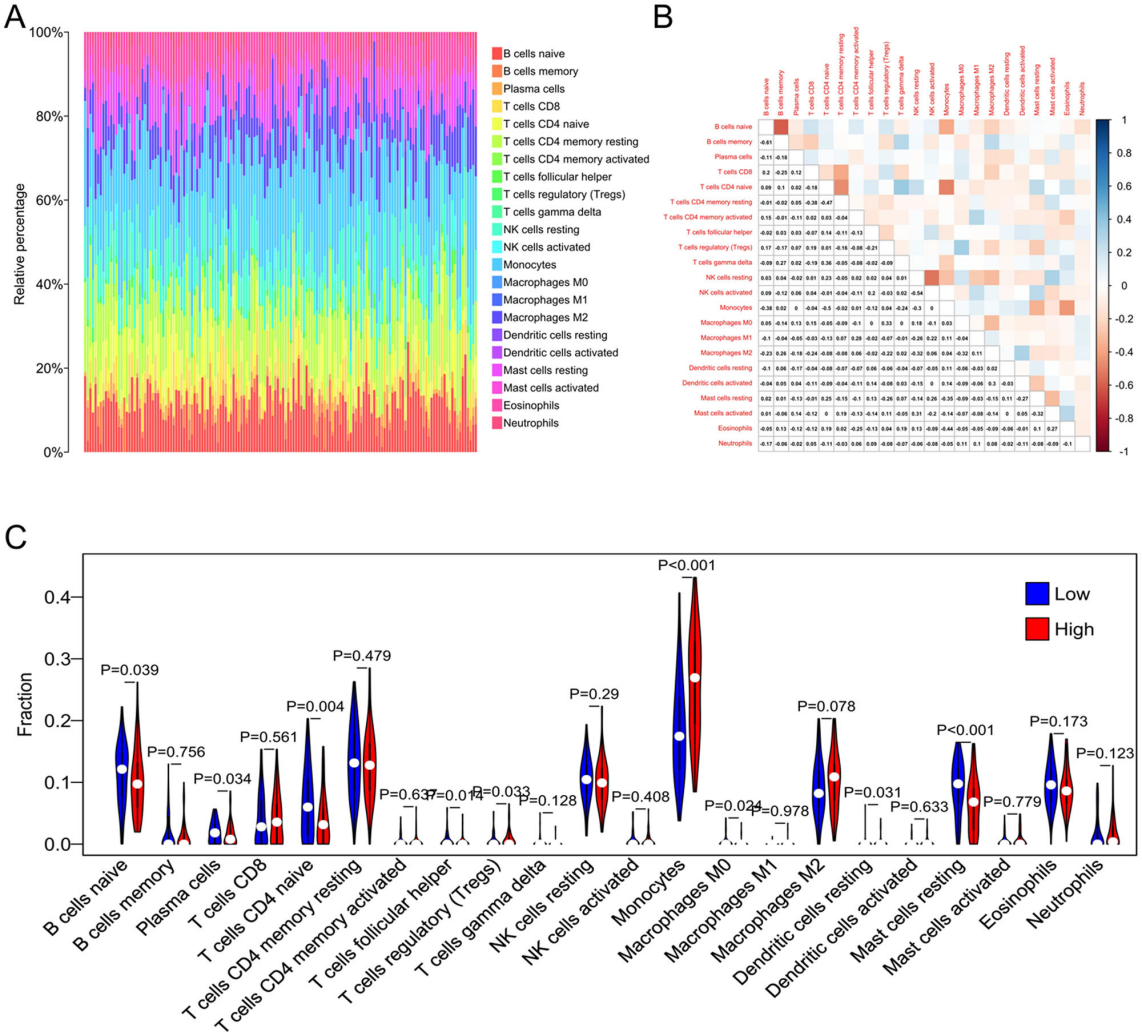
SFXN3 is linked to immune cell infiltration in AML analyzed by CIBERSORT. **A** The abundance of the 22 types of immune cells in AML. **B** The correlation among the 22 immune cells. **C** The relationship between SFXN3 and the infiltration of the 22 immune cells analyzed by spearman method

The prognosis of AML is closely correlated with the infiltration of immune cells. Therefore, we assessed the correlation between SFXN3 and the infiltration of immune cells. The relative levels of the 22 types of immune cells in the CGGA datasets were evaluated by cibersort. We firstly analyzed the total status of these cells in AML tissues. The results of the bar plot exhibited the abundance of these cells in AML tissues which demonstrated that most of the dominant cell types belongs to the innate immune system, while adaptive immune cells, T and B cells, are not abundant (Fig. 5A). Besides, the correlation among the 22 immune cells were analyzed (Fig. 5B). Then, we explored the relationship between SFXN3 and the infiltration of the 22 immune cells via spearman method. The violin plot displayed that the level of SFXN3 are negatively related to the infiltration of memory B cells, eosinophils, resting mast cells, naive CD4^+^ T cells and gamma delta T cells (Fig. 5C).

**Fig. 6:**
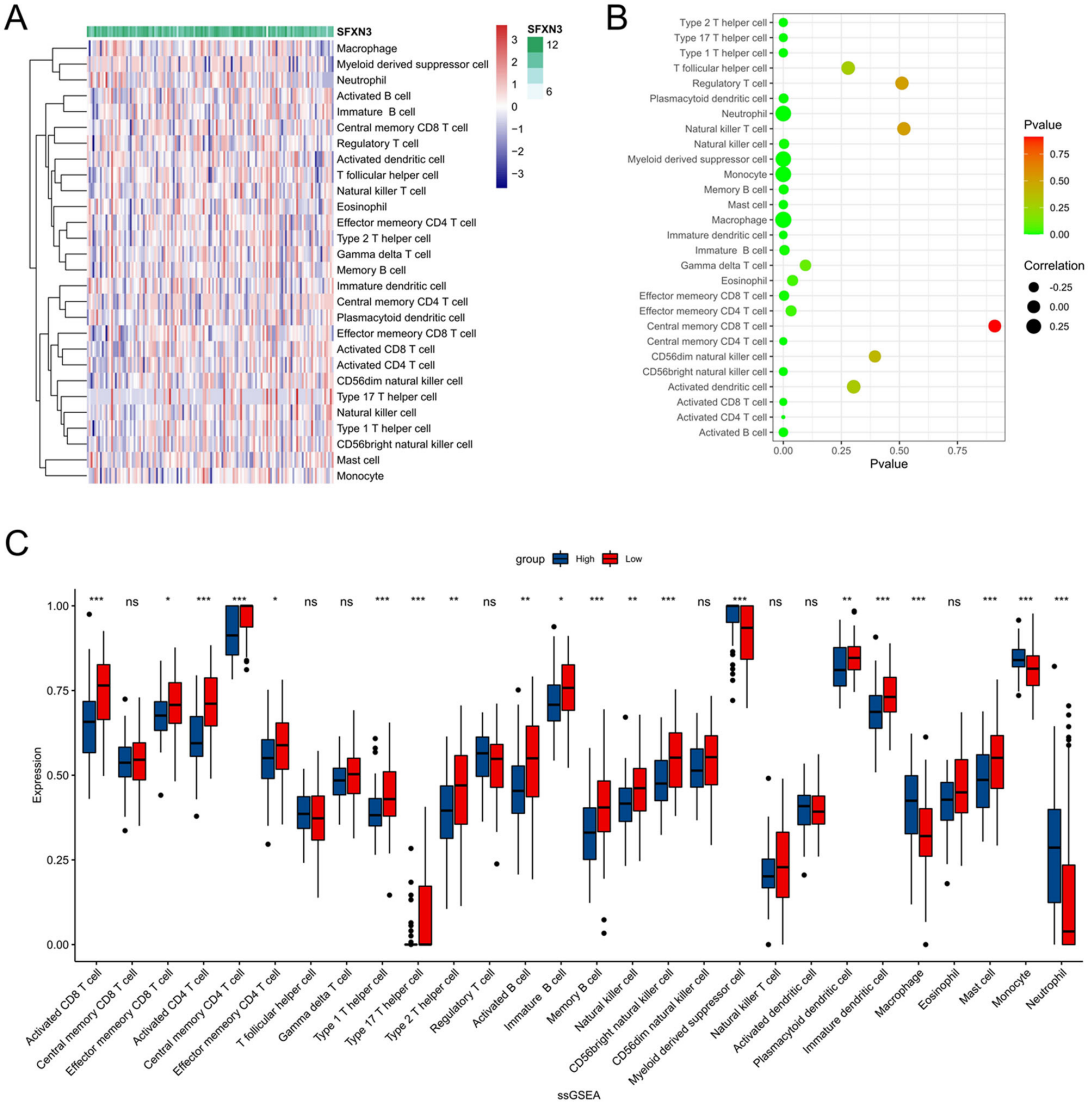
SFXN3 is linked to immune cell infiltration in AML analyzed by ssGSEA. The correlation between SFXN3 and 28 types of immune cells in AML displayed through a heatmap (**A**), bubble pot (**B**) and bar plot (**C**)

Furthermore, we explored the correlation between SFXN3 and 28 types of immune cells from the CGGA dataset through ssGSEA algorithm using Pearson method (Fig. 6A). We divided these AML samples into SFXN3 high and low expression groups and display the results using a bubble plot. The results indicated that SFXN3 is positive correlated with the infiltration of neutrophil, monocyte/macrophage and myeloid derived suppressor cell (MDSC), but negative related to most of the 28 cells, such as CD8^+^T cells, B cells and NK cells (Fig. 6B). Besides to the bubble plot, a bar plot was also draw to exhibit the results (Fig. 6C).

Although, the results yielded from the cibersort and ssGSEA analyses exist difference, which may largely attribute to the algorithm, we can find that SFXN3 is related to a immunosuppressive status.

## Discussion

AML is a serious threat to human health [3, 5], and about 30% of patients with non-acute promyelocytic leukemia can expect to survive long term [2, 16]. However, for these patients, a good quantitative tool for predicting survival time remains need to be explored [17, 18]. In this research, we firstly analyzed the level and prognostic value of SFXN3 in pan-cancer, especially in AML, based on the data downloaded from TCGA database. The results indicated that SFXN3 level increased in AML, and its increased expression is an indicator of poor prognosis. This result is similar with previous studies, which reported that SFXN3 as a negative prognostic marker for non-M3 AML, head and neck cancer, and oral squamous cell carcinoma [15, 19, 20].

Although the expression and clinical value of SFXN3 have been explored in rare tumors, its function in AML remains largely unclear. In order to investigate the biological function, SFXN3 siRNA was used to transfect cells, the results presented that silencing SFXN3 expression can effectively suppressed cells proliferation, and the G1/S arrest may be the cause. Besides, silencing SFXN3 expression also effectively enhanced the prognosis of cells and the increased caspase-3 activity may be the reason. Cell cycle is controlled by cyclin and their corresponding cyclin-dependent kinases. For the transition from G1 to S stage, the controlled cyclin proteins are cyclin D and E (CCND and CCNE). The cyclin-dependent kinases connecting to CCND are CDK4 and CDK6, while connecting to CCNE is CDK2. Therefore, we measured the levels of these markers and found that CCND1 is negatively regulated by SFXN3. Furthermore, we measured the expression of typical reported upstream markers of CCND1, and NFκB1 was screened out. According to these results, we concluded that SFXN3 could inhibit the apoptosis and promote the proliferation of AML cells, and NFKB1-CCND1 pathway may involve in this regulation.

We also explored the potential effect of SFXN3 in affecting the immune cells in the tumor micro-environment via CIBERSORT and ssGSEA. The results indicated that SFXN3 can reduce the infiltration of NK and CD^8+^ T cells. Also, SFXN3 can reduce the level of active CD^4+^ T cells, Th1, Th2 and Th17 cells, however, SFXN3 does not affect the level of Treg cells. The main reason may be that SFXN3 sustains the stability of Treg cells by blocking the differentiation of CD^4+^ T cells in other directions. Taken together, we hypnotized that SFXN3 may result in the immunosuppressive state in AML, which is required to be validated in our further study.

To our knowledge, this is the first comprehensive bioinformatics analysis to elucidate the potential function and prognostic value of SFXN3 and its association with immune infiltration. However, there are limitations to the study. First, SFXN3 levels were not detected in patients with AML. Secondly, the mechanism of SFXN3 action needs further molecular experiments. Finally, the effect of SFXN3 on the infiltration of immune cells in AML needs to be verified in different type AML cell lines in the future.

## Conclusion

SFXN3 is highly expressed in AML, and the increased SFXN3 expression is associated with decreased OS in AML. Next, SFXN3 increases the proliferation and decreases the prognosis of AML cells, and NFKB1-CCND1 pathway may attribute to this function. Finally, SFXN3 may lead to the immunosuppressive state in AML. Our study provides novel insights into the treatment and prognostic prediction of AML.

## Material and Methods

### Data Source and Expression Analysis

Pan-cancer data including 31 types of cancers were obtained from The Cancer Genome Atlas (TCGA) database. The expression of SFXN3 in these cancer were analyzed via Gene Expression Profiling Interactive Analysis (GEPIA), an online database, and the threshold was set as |log^fold change (FC)^ | ≥ 1 and p value < 0.05. GEPIA Kaplan-Meier curve was used to analyze the relationship between SFXN3 level and the OS of AML patients in TCGA.

### Immune Cells and Bioinformatic Analysis

The CIBERSORT and single sample gene set enrichment analysis (ssGSEA) were employed to perform immune related analysis. The expression matrices of 22 types of immune cells were deconvolved by using CIBERSORT method and linear support vector regression principle to explore the differences of immune cells in AML. Subsequently, we screened out immune cells with significant differences in immune infiltration in patients with AML, and analyzed the correlation between immune cells and SFXN3 by spearman method. The abundance of 28 types of immune cells were analyzed. Subsequently, we screened out immune cells with significant differences in immune infiltration in patients with AML, and analyzed the correlation between immune cells and SFXN3 by Pearson method.

### Cell Culture and Transfection

Human leukemia cell lines (OCI-AML2, OCI-AML5, MEG-01, KU812, K562, SKM-1 and KG-1a) were cultured in Dulbeco Modified Eagle Medium (DMEM) containing 10% fetal bovine serum, 1% L-glutamine, 1% penicillin, and 1% streptomycin in a 5% CO_2_ humidified incubator at 37°C. MEG-01 and SKM-1 cells were transfected with Lipofectamine 3000 (Invitrogen, Carlsbad, USA) and anti-SFXN3 siRNA (GeneCodex, China) according to the manufacturer’s instructions. MEG-01 and SKM-1 cells were collected after 48 h for subsequent experiments.

### Reverse-transcription and Quantitative Real-time PCR (qRT-PCR)

According to the manufacturer’s instructions, Trizol reagent (Takara, Japan) was used to lyse the cells and extract total RNA. The RT Premix Kit (Takara Bio Inc.) is for reverse transcription. The reaction was carried out at 37 °C for 15 min and 85 °C for 5 s. qRT-PCR was performed using a real-time PCR system (Bio-Rad Laboratories, Hercules, CA, USA) and the results were calculated based on 2^-∆∆Ct^ method.

### Western Blotting

CCND1 and NFKB1 protein levels in MEG-01 cells were determined by western blotting according to the manufacturer’s instructions. In brief, cells from different groups were collected, then washed them twice with PBS, and lysed in pre-cooled 2× lysis Buffer, and centrifuged at 4°C, 12000×g for 15 min. The protein concentration was detected using the BCA kit (Beyotime Biotechnology, Shanghai, China). 30 μg protein was uploaded and separated by 12% SDS-PAGE, then transfected onto PVDF membrane. Primary antibodies were added and incubated with the PVDF membrane sealed with blocking solution (TBST solution containing 5% skim milk) at 4 °C overnight. The membrane were washed with TBST for three times. The secondary antibody (goat anti-Mouse IgG (H + L) or goat anti-rabbit IgG (H + L)) was added and incubated at room temperature for 2 h, then washed the membrane 3 times with TBST. Anti-CCND1 antibody (1:500; CST), anti-NFKB1 antibody (1:500; Sigma), GAPDH (1:1000; Affinity) and Goat Anti-Rabbit IgG (H + L) HRP (1:2000; Affinity) were used in this study. Protein bands were developed using enhanced chemiluminescence (ECL, ThermoFisher Scientific, USA). Image J software was used for analysis and relative quantification.

### CCK-8 Assay

CCK8 assay was performed as the manufacturer’s instructions. Briefly, AML cells (1×10^3^) transfected with siSFXN3 or siNC were incubated with CCK8 reagent (Beyotime Biotechnology, Shanghai, China) at 37 °C for 1 h. Then, the amount of cells was determined by measuring the absorbance at 450 nm for consecutive five days.

### Cell Cycle and Apoptosis by Fluorescence-activated Cell Sorting (FACS) Analysis

Cell cycle and apoptosis of AML cells was measured through FACS. AML cells were seeded into 6-well plates and transfected with siSFXN3 or siNC. After 72 h, AML cells were collected. For cell apoptosis measurement, after different treatment, cells were incubated with 5 μL FITC and 5 μL PI staining reagents for 5 min in the dark. For cell cycle detection, different treatment cells were incubated with staining buffer for 10 min in the dark. Finally, the proportion of apoptotic AML cells and the cell cycle of AML cells were analysis via FACS by flow cytometry (Thermo Fisher Scientific, USA).

### Caspase 3 Activity Assay

Caspase 3 activity was determined by caspase 3 activity assay kit (Beyotime Biotechnology, Shanghai, China). AML cells were seeded into 6-well plates and transfected with siSFXN3 or siNC. After 72 h, AML cells were collected and cracked for 15 min. Caspase 3 activity was detected based on the manufacturer’s instructions.

### Xenograft Experiments

Female BALB/c nude mice with 4-week old were subcutaneously injected with shSFXN3 or shNC MEG-01 cells (5×10^6^ cells in 100 μL PBS) to construct xenograft mice model. The volume of the tumors were measured every three days after the tumors formulated. The weight of the tumors were measured after the mice scarified. All animal experiments were performed based on the Guide for the Care and Use of Laboratory Animals of the National Institutes of Health [National Research Council (US) Institute for Laboratory Animal Research, 1996] and were approved by the Ethics Committee of The First Affiliated Hospital of Anhui Medical University.

### Statistical Analysis

Statistical analysis was performed using GraphPad Prism (San Diego, USA). Receiver-operating characteristic curves was generated to determine the diagnostic value of SFXN3 genes. Difference between two groups was compared by *t*-test. P < 0.05 was considered significant.
